# 2022 Health Behaviour in School-aged Children topic-specific reports

**DOI:** 10.24095/hpcdp.46.2.06

**Published:** 2026-02

The 2022 Health Behaviour in School-aged Children (HBSC) is a national cross-sectional study of over 26000 students in Grades 6–10 from over 300 Canadian schools. Queen’s University, in collaboration with national experts, has developed four topic-specific national reports offering focussed insights into the 2022 HBSC.

## Highlights

- **Food insecurity** impacts 1 in 5 students, with higher rates among families of lower socioeconomic status. Youth experiencing food insecurity were less likely to meet nutrition, sleep and physical activity recommendations. They were also more likely to report weekly health symptoms (i.e. headaches, irritability and feeling low) and lower life satisfaction.
- **Adherence to 24-hour movement behaviour recommendations** continues to decline. Only 38% met daily physical activity recommendations in 2022 (down from 59% in 2018). Screen time (averages of 4.5–5.5 hours per day) exceeded recommendations and adherence to sleep guidelines fell to 58%. Fewer than 1 in 10 youth met all three recommendations, with gender-diverse youth reporting the lowest proportion meeting recommendations.
- **Online risk-taking** is common among youth, with 40% having friended strangers, 40% having followed social media diets or exercise plans and 30% having participated in “internet challenges.” Smaller proportions of youth bought drugs online (7%) or met online strangers (5%). These behaviours were linked to poor mental health, especially loneliness and low life satisfaction.
- **Adolescent dating violence** impacted 36% of youth who were in a relationship, with higher rates among transgender and genderdiverse youth (44%) and cisgender girls (42%). Strong family, peer and teacher support reduced the risks.

To access or download the national reports, visit the Queen’s University HBSC web page. 

